# Morphological and Molecular Identification of *Tritrichomonas mobilensis* in Captive Ring-Tailed Lemurs (*Lemur catta*)

**DOI:** 10.3390/vetsci12121188

**Published:** 2025-12-12

**Authors:** Chaowu Fu, Yiheng Ma, Rao Li, Decheng Wang, Ziguo Yuan, Yurong Yang

**Affiliations:** 1 Laboratory of Herbivore Nutrition, College of Animal Science and Technology, Henan Agricultural University, Zhengzhou 450002, China; 2 Laboratory of Veterinary Pathology, College of Veterinary Medicine, Henan Agricultural University, Zhengzhou 450002, China; 3 Hubei Key Laboratory of Tumor Microenvironment and Immunotherapy, College of Basic Medical Sciences, China Three Gorges University, Yichang 443002, China; 4 Key Laboratory of Zoonosis Prevention and Control of Guangdong Province, College of Veterinary Medicine, South China Agricultural University, Guangzhou 510642, China

**Keywords:** *Tritrichomonas mobilensis*, ring-tailed lemur, squirrel monkey, non-human primates, isolation

## Abstract

In September 2024, an acute hemorrhagic diarrhea occurred in a colony of captive ring-tailed lemurs (*Lemur catta*) housed adjacent to an enclosure of squirrel monkeys in Henan Province, China. Three of the ring-tailed lemurs (1-year-old females) died, and necropsy revealed severe cecal hemorrhage. Histopathology demonstrated extensive mucosal necrosis, hemorrhage, and inflammatory cell infiltration in the cecum, with parasite adhesion and invasion into the crypts. Microscopic examination and PCR targeting the ITS1-5.8S-ITS2 region (347 bp) confirmed the presence of *T. mobilensis* in the deceased lemurs’ intestinal contents and in feces from 40.0% (4/10) of the squirrel monkeys. Ultrastructural analysis (SEM/TEM) confirmed that the isolated parasites exhibited the morphology and key features of *T. mobilensis*, including prominent peripheral hydrogenosomal vesicles. Treatment with metronidazole and supportive care led to the recovery of the surviving lemurs. Post-treatment fecal exams of ring-tailed lemur were negative for *T. mobilensis*. This report provides the first evidence of *T. mobilensis* infection in ring-tailed lemurs, indicating cross-species transmission from squirrel monkeys to lemurs, likely facilitated by keeper-mediated mechanical transmission via tools and footwear that were used in both enclosures.

## Simple Summary

*Tritrichomonas mobilensis* is an intestinal protozoan parasite whose natural host is squirrel monkeys (*Saimiri* spp.); infection is typically asymptomatic. *T. mobilensis* infection has also been demonstrated in tree shrews and gibbons. However, its pathogenicity and prevalence in other species remain largely unknown. This report provides evidence of *T. mobilensis* infection in ring-tailed lemurs; *T. mobilensis* exhibited significant pathogenicity (hemorrhagic enteritis, mortality) in this novel, accidental host. Our findings highlight the potential risk *T. mobilensis* poses to non-natural hosts in shared environments, including humans.

## 1. Introduction

Parabasalid protists in the intestines are prevalent in insects (termites), reptiles, birds, and mammals, including human and nonhuman primates (NHPs) [1]. The source of the samples impacts the positivity rates, and the diagnostic methods alter the sensitivity and specificity of the test, thereby influencing the estimation of parabasilid infection in hosts. The high prevalence of parabasilids in the digestive tracts of monkeys, dogs, raccoon dogs, humans, and wild rodents was observed; they were 47% (28/60), 27% (69/252), 26% (100/389), 9% (13/142) and 4% (,19/510), respectively [2,3,4]. In general, intestinal parabasilids impact host immunity and gut microbiome ecology and are rarely responsible for lesions. Nevertheless, reports of lesions and clinical manifestations induced by parabasilids have been reported in humans, rodents, and NHPs [5,6,7,8,9]. *Pentatrichomonas hominis*, *Trichomitus batrachorum*, *Dientamoeba fragilis*, and *Tritrichomonas mobilensis* have been detected in NHPs [7,8,9,10].

*Tritrichomonas mobilensis* is an intestinal protozoan parasite belonging to the phylum Parabasalia, family Tritrichomonadidae [9,10,11]. Like other members of the genus Tritrichomonas, *T. mobilensis* has a parabasal body, three anterior flagella, an undulating membrane forming a posterior flagellum, and an axostyle [10]. Its natural host is squirrel monkeys (*Saimiri sciurea* or *Saimiri boliviensis*) [11,12,13]. While *T. mobilensis* infection has been demonstrated in tree shrews and gibbons [9], it remains unclear whether this parasite can infect other species.

We describe an outbreak of acute intestinal trichomoniasis in a colony of captive ring-tailed lemurs (*Lemur catta*) in China, representing the first report of *T. mobilensis* infection of this species.

## 2. Case Description

### 2.1. Samples

**Figure 1:**
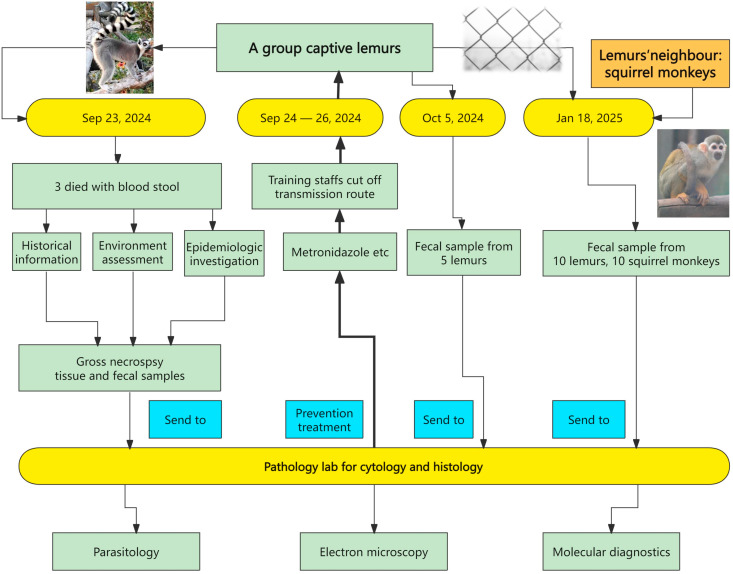
The timeline of the pathological process and sampling in lemurs and squirrel monkeys.

On 23 September 2024, a group of captive ring-tailed lemurs at a wildlife park in Henan Province, China, developed acute diarrhea and hematochezia. Three 1-year-old female lemurs exhibited severe clinical symptoms and died. Notably, the ring-tailed lemurs’ enclosure was next to an enclosure occupied by asymptomatic squirrel monkeys, separated only by a wire fence. Their care was managed by the same keeper, who did not change his shoes or use different tools when working in the two enclosures. The intestines with bloody stool were preserved in a plastic sealed bag at 4 °C, without the use of any chemical preservatives. Each organ was stored in its own sealed bag separately, but the scissors and forceps were not replaced during the necropsy. Tissue samples from the deceased lemurs (intestines, heart, liver, spleen, lungs, and kidneys) were collected by zoo veterinarians and submitted to the Laboratory of Veterinary Pathology, Henan Agricultural University (Zhengzhou, Henan, China) for histopathological analysis and etiological investigation (Figure 1). On 5 October 2024, five ring-tailed lemurs’ fecal samples from the same wildlife park were collected by zoo veterinarians and submitted to the lab to check for possible pathogens. To identify the etiology causing diarrhea in ring-tailed lemurs and determine the transmission source, the third sampling in the same wildlife park was conducted on 18 January 2025. Fecal samples from lemurs (*n* = 10) and the squirrel monkeys (*n* = 10) were collected by the first two authors.

### 2.2. Coproparasitological and Histopathological Analysis

To explore for the possible bacteria and parasites that may cause bloody stools, direct smears, Gram staining, and Giemsa staining of the intestinal contents of the three dead lemurs were processed and observed under an Olympus BX43 light microscope (Olympus Corporation, Tokyo, Japan) with magnification of 400 or 1000. Each animal undergoes observations and statistics covering the areas of two coverslips (18 mm × 18 mm). Positive samples were further subjected to PCR to confirm the pathogen. To explore the pathological lesions, the tissue samples from ring-tailed lemurs were fixed with 10% neutral-buffered formalin solution, then processed using routine histological techniques and stained with hematoxylin and eosin (H&E). The sections were observed under a microscope to evaluate the lesions and speculate on the etiology of the lesions according to James (2022) [14].

### 2.3. DNA Extraction and PCR

To specifically identify *Tritrichomonas* spp. by molecular means, DNA was extracted from the collected tissue and fecal samples using a DP304 DNA extraction kit (Tiangen Biotec Company, Beijing, China). The internal transcribed spacer 1 (ITS1)––5.8S ribosomal DNA––internal transcribed spacer 2 (ITS2) region was amplified using the following primer pair [15]: TFR3 (forward primer 5′-CGG GTC TTC CTA TAT GAG ACA GAA CC-3′) and TFR4 (reverse primer 5′-CCT GCC GTT GGA TCA GTT TCG TTA A-3′). The 50 µL reaction mix included 25 µL of GDSBio 2× PCR Mix (Guangzhou, China) and 2 μM of each primer. Amplification was performed on a Bio-Rad T100 thermal cycler (Hercules, CA, USA) with the following conditions: initial denaturation at 94 °C for 5 min, 40 cycles of denaturation at 94 °C for 30 s, annealing at 67 °C for 30 s, and extension at 72 °C for 90 s, and a final extension step of 15 min at 72 °C. Negative (*Toxoplasma gondii* RH DNA) and positive (feline *Tritrichomonas foetus* DNA) controls were included in each PCR reaction. The amplified products were then analyzed by gel electrophoresis. The PCR products were sent to Sangon Biotech Co. (Shanghai, China) for bidirectional sequencing using both amplification primers (TFR3 and TFR4). Sequence assembly and phylogenetic analysis were carried out using SeqMan Pro 7.1.0 (DNASTAR, Madison, WI, USA) and MEGA 7.0 [16], with Clustal W multiple alignment. Subsequently, a phylogenetic tree was constructed using the Neighbor Joining (NJ) method and the Tamura 3-Parameter model with 1000 bootstrap replications.

### 2.4. Culture of Tritrichomonas spp. and Electron Microscopy

To perform ultrastructural analysis of the parasites, the *Tritrichomonas* spp. were purified from intestinal contents or feces by differential centrifugation and cultured in vitro at 37 °C in Roswell Park Memorial Institute (RPMI) 1640 medium supplemented with 10% fetal bovine serum (FBS) [17]. The culture medium was renewed every 24 h, and the inoculation ratio was 1:10. Single parasites were isolated from cultures in the logarithmic phase of growth by limiting dilution or micromanipulation [18] and then expanded into clonal populations by further culturing in RPMI 1640 + 10% FBS. *Tritrichomonas* spp. isolates were frozen in liquid nitrogen with dimethylsulfoxide, 10% RPMI1640 medium, and FBS (2:3:5). The cultures were centrifuged (500× *g* for 10 min) and fixed in 2.5% glutaraldehyde at 4 °C for 2 h, rinsed with PBS, and then postfixed in 1% osmium tetraoxide (*w*/*v*) at 4 °C for 1 h. The parasites underwent progressive dehydration using 70%, 80%, 90%, 95% and 100% alcohols for 10 min each at 4 °C and were subjected to drying under a critical point, mounted on aluminum stubs, and sputtered with gold. Then, the surface of parasites was examined at 25 KV with a scanning electron microscope (SEM) (Hitachi SU 3500, Tokyo, Japan). Furthermore, parasites were fixed in glutaraldehyde for 2 h at 4 °C, postfixed in 1% osmium tetroxide (*w*/*v*) for 1 h at 4 °C, and then dehydrated in a graded series of alcohols and embedded in Epon812 epoxy resin. Ultrathin sections (thickness: 600–900 Å, 65 nm) were stained with uranyl acetate and plumbum citrate and observed under the transmission electron microscope (TEM) (Hitachi 7800, Tokyo, Japan). A feline isolated strain of *T. foetus* maintained in our laboratory was also observed by TEM for ultrastructural comparison.

## 3. Results

**Figure 2:**
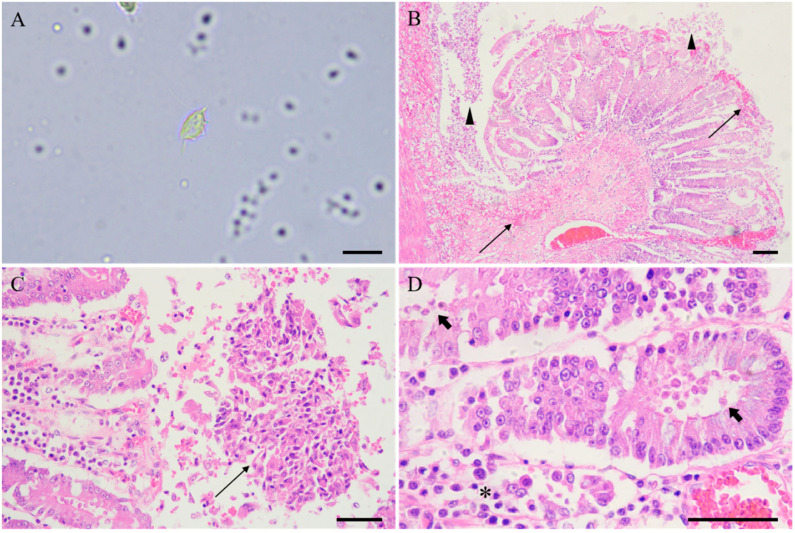
Light microscopy images of *Tritrichomonas* mobilensis and histopathology of ring-tailed lemurs. (**A**) *T. mobilensis* was observed in the cecal contents, with clearly visible anterior flagella, undulating membrane, axostyle, and recurrent flagellum, smear, unstained, ring-tailed lemur. (**B**) Acute diffuse necrosis of the intestinal mucosa was observed. Note the necrosis and disintegrating shedding of mucosal epithelial cells (arrowheads), with widespread hemorrhage (thin arrows), H&E. (**C**) Numerous *T. mobilensis* were observed adhered to the surface of the intestinal mucosa, as well as detached, necrotic mucosal epithelial cells (thin arrows), H&E. (**D**) *T. mobilensis* had invaded the intestinal crypts (thick arrows), and lymphocytes and plasma cells had infiltrated the lamina propria (asterisk), H&E. Bar: (**A**) 10 µm, (**B**) 100 µm, (**C**) 50 µm and (**D**) 50 µm.

Autopsy revealed intestinal hemorrhage and relatively severe lesions in the cecum, while no significant gross pathological changes were observed in other organs. Direct smears of the intestinal contents of the three dead lemurs (Figure 2A) and feces from two of the ten squirrel monkeys showed a substantial amount of *Tritrichomonas* spp. with consistent morphology. *Tritrichomonas* spp. isolates from three dead lemurs were all successfully obtained in 10% RPMI1640 medium with 10% FBS. They were oblong, pear-shaped, or round, with a length of about 12 ± 1.2 μm and a width of 5.5 ± 0.6 μm. They exhibited three anterior flagella, a trailing flagellum, and an undulating membrane extending over the entire side of the parasite’s body, which matches *Tritrichomonas* spp. morphology.

Consistent with the fact that gross lesions were only observed in the cecum of the ring-tailed lemur upon autopsy, no significant histopathological changes were observed in the heart, liver, spleen, lung, or kidney of the lemurs. However, numerous *Tritrichomonas* spp. (5 × 10^6^/mL) were found in the lumen of the cecum, where they had adhered to the intestinal mucosal epithelium and invaded the crypts. Histopathological changes in the affected cecum included extensive hemorrhage in the mucosal layer, disintegrating shedding of mucosal epithelial cells, cellular necrosis in the crypts, and infiltration of inflammatory cells (mainly lymphocytes and plasma cells) into the lamina propria (Figure 2B–D). Other parasites were not detected either in the coproparasitological examination or in the histopathology observation.

**Figure 3:**
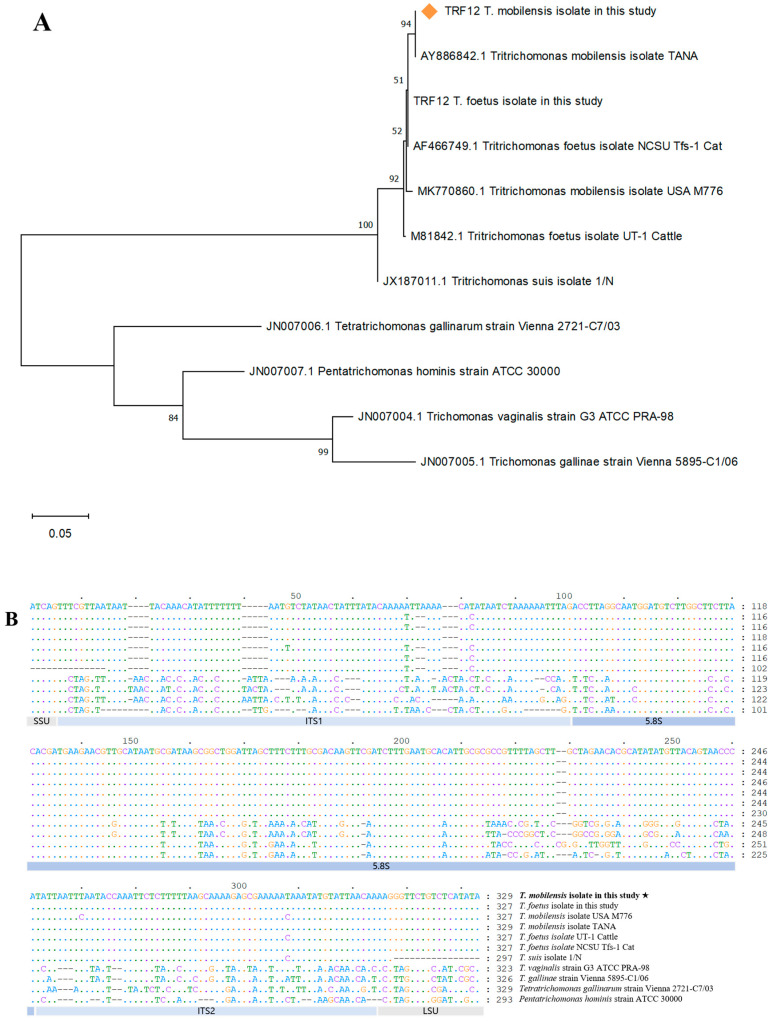
Molecular analysis of the *Tritrichomonas mobilensis* isolate from the ring-tailed lemurs and other trichomonads. (**A**) Phylogenetic tree constructed based on the ITS1-5.8S-ITS2 sequences, the orange square represents the isolate in this study; (**B**) Sequence alignment of the ITS1-5.8S-ITS2 region, the star sign represents the isolate in this study.

Single products of the expected size (347 bp) were amplified from tissue samples of the dead ring-tailed lemurs (cecum, heart, liver, spleen, lungs, and kidneys), which identify *Tritrichomonas* spp. by PCR. Four out of ten squirrel monkey feces samples also showed nucleic acid of *Tritrichomonas* spp. The ITS1-5.8S-ITS2 sequences from the isolated pathogens clustered naturally with *Trichomonas* spp. members and were identical to those of the *T. mobilensis* TANA strain (Figure 3), demonstrating that the pathogen was indeed *T. mobilensis*. Other possible bacteria with pathogenic genes or flagella were not detected by PCR from the intestines of the dead ring-tailed lemurs.

The *T. mobilensis* parasite could be isolated from the intestinal contents of the dead ring-tailed lemurs in RPMI 1640 and FBS medium, but difficulty isolating it from asymptomatic squirrel monkey feces samples. Further, after the culture medium was renewed 2–3 times, the number of bacteria decreased, and the growth of *T. mobilensis* became poor.

**Figure 4:**
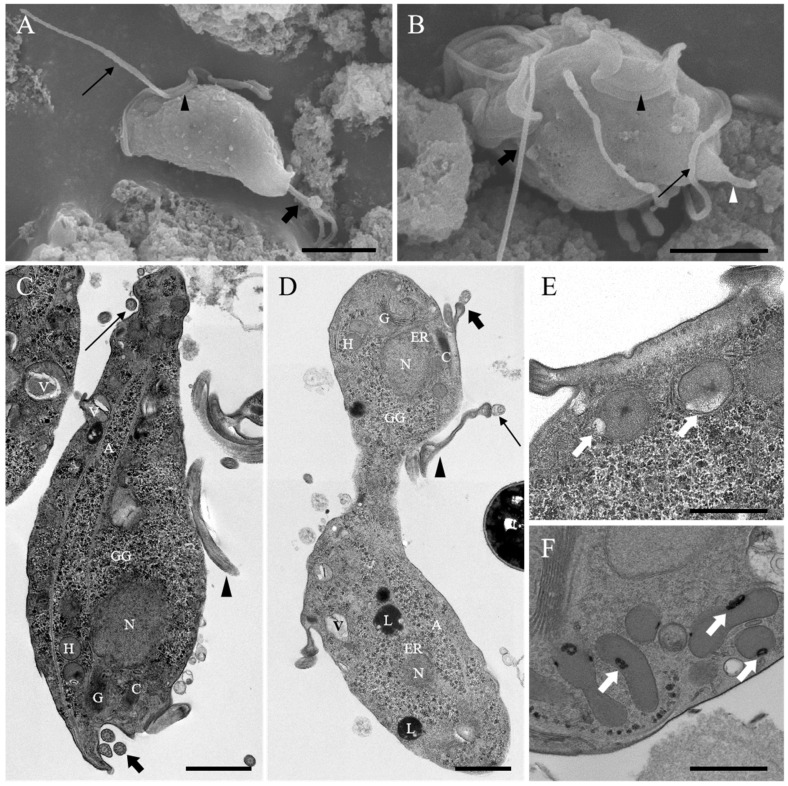
Scanning electron microscopy (SEM) and transmission electron microscopy (TEM) images of *Tritrichomonas* mobilensis (ring-tailed lemur) and *Tritrichomonas* foetus (cat). (**A**,**B**) SEM of *T. mobilensis* cultured in vitro. Three anterior flagella (thick arrow), an undulating membrane (black arrowhead) reaching the posterior end of the body, a recurrent flagellum (thin arrow), and the axostyle (white arrowhead) are visible. (**C**,**D**) TEM of *T. mobilensis* cultured in vitro. A cell undergoing mitosis can be seen in (**D**). The parasite had a spindle-shaped body and exhibited typical tritrichomonad morphology features, such as three anterior flagella (thick arrow), an undulating membrane (black arrowhead), a recurrent flagellum (thin arrow), the nucleus (N), several hydrogenosomes (H), the Golgi complex (G), the rough endoplasmic reticulum (ER), the striated costa (C), the axostyle (A), abundant glycogen granules (GG), food vacuoles (V), and lysosome-like structures (L). (**E**,**F**) TEM images of *T. mobilensis* (**E**) and *T. foetus* (**F**) hydrogenosomes. The organelles in both species presented a single peripheral vesicle (white arrows), but the peripheral vesicles in *T. mobilensis* were larger, more prominent, and translucent, whereas those in *T. foetus* were flattened, exhibited high-density features, and had a more elongated, fava bean–like shape. Bar: (**A**,**B**) 5 µm, (**C**,**D**) 2 µm, (**E**,**F**) 1 µm.

Surface analysis of the *Tritrichomonas* spp. by SEM showed three anterior flagella, an undulating membrane reaching the posterior end of the body, a recurrent flagellum, and an axostyle (Figure 4A,B). TEM analysis of the intracellular region primarily showed the nucleus, several hydrogenosomes, the Golgi complex, the rough endoplasmic reticulum, the striated costa, the axostyle, abundant glycogen granules, food vacuoles, and lysosome-like structures (Figure 4C,D). Furthermore, we observed *T. mobilensis* mitosis by TEM (Figure 4D). The anterior regions of the two daughter cells separated first and moved in opposite directions, with the posterior region remaining connected during mitosis. In addition, while the hydrogenosomes contained single peripheral vesicles in both *T. mobilensis* and *T. foetus*, in *T. mobilensis* these vesicles were larger, more prominent, and translucent, while in *T. foetus* they were flattened, exhibited high-density features, and had a more elongated, fava bean–like shape (Figure 4E,F).

Symptomatic ring-tailed lemurs were treated with intramuscular injections of gentamicin (5 mg/kg body weight) and sulfonamide (20 mg/kg body weight), in conjunction with oral administration of montmorillonite powder (50 mg/kg body weight), 60 mg/kg body weight of metronidazole, vitamin A (200 IU/monkey/day), and vitamin E (10 IU/monkey/day) for three days (24–26 September 2024) (Figure 1). Concurrently, the ring-tailed lemurs and squirrel monkeys were prevented from encountering each other by reinforcing the separation between their adjacent enclosures, and feces within both enclosures were removed daily. All ring-tailed lemurs recovered after three days of treatment. The staff were subsequently trained to use separate tools, gloves, and shoes for each animal area and to mark them with different colors for distinction. Two weeks later (5 October 2024), a follow-up ring-tailed lemur fecal examination performed by PCR and direct smear revealed no *Tritrichomonas* spp. infection (0/5) after treatment (Figure 1).

## 4. Discussion

*Tritrichomonas mobilensis* was isolated from the intestine of the deceased ring-tailed lemurs, and its identity was confirmed by ultrastructural observation and molecular characterization. These morphological features are consistent with those reported in a previous study [10]. The infected ring-tailed lemurs exhibited symptoms including diarrhea and hematochezia. Histopathological examination showed that *T. mobilensis* was highly pathogenic in the ring-tailed lemurs, causing extensive necrosis and hemorrhage of the posterior part of the intestinal mucosa, as well as a marked inflammatory response. After treatment, the surviving ring-tailed lemurs showed significant improvement in symptoms with treatment and recovered after three days. In this study, the feces of the staff were not tested for *T. mobilensis*, and none of them showed bloody stools or diarrhea. However, they were suggested for the *T. mobilensis* test in the local hospital, washed thoroughly after work, drank boiled water, and ate cooked food.

Enteric bacteria (*Vibrio fetus*, *Salmonella enterica*, *Clostridium perfringens*, *Escherichia coli* O157:H7, and spirochetes) and toxication may produce similar syndromes of acute hemorrhagic enteritis in primates or food animals, although pathophysiology and molecular pathogenesis may vary [19,20,21,22,23]. However, pathological and etiological methods can help determine primary etiology or secondary etiology for hemorrhagic enteritis.

Squirrel monkeys are the natural host of *T. mobilensis*, in addition to tree shrews and gibbons [6,7,8,9], which have not been reported to infect other mammals in the wild. In laboratory colonies, newborn squirrel monkeys do not carry this parasite, but most squirrel monkey infants show evidence of trichomoniasis at 2 to 4 weeks of age, and 100% are infected by 8 weeks of age [24]. One study reported that 77.2% (156/202) of tree shrews in a laboratory colony were infected with *T. mobilensis*, and age group analysis suggested early postnatal infection, with an almost linear, rapid increase in the percentage of infected animals from 7.6% in the neonatal group to approximately 90% in animals older than 1 year, and then decreased after 1 year old, about 74% in tree shrews more than 4 y [9]. The researchers hypothesized that the infection was acquired through contact with squirrel monkeys in the laboratory. This peak age of occurrence in tree shrews is consistent with the onset of the current case of lemurs (one year old). In the present study, fecal samples from 40.0% (4/10) squirrel monkeys were positive for *T. mobilensis* by PCR, while parasites were detected by microscopy in 20.0% of the fecal samples; however, none of the infected squirrel monkeys showed any clinical symptoms. Given the proximity of the ring-tailed lemurs’ enclosure to that of the squirrel monkeys, the natural host of *T. mobilensis*, we hypothesize that the lemurs acquired the parasite via the fecal-oral route. It is likely that the keeper played a non-trivial role in the mechanical transmission of this parasite.

Regarding symptomology, the ring-tailed lemurs infected with *T. mobilensis* exhibited diarrhea and hematochezia, and some even died, but the fecal-positive squirrel monkeys were healthy. This suggests that squirrel monkeys and *T. mobilensis* have developed a symbiotic relationship over a long period of host–parasite co-evolution. However, because of this host specificity, the parasite triggers serious consequences in species other than its natural hosts [25]. A previous study reported that *T. mobilensis* injected subcutaneously into mice invaded the internal organs and was ultimately lethal [6]. *Tritrichomonas* spp. do not normally infect humans; they are considered opportunistic protozoan pathogens in humans [26]. However, two cases of lethal peritonitis and meningoencephalitis triggered by *T. foetus* infection in immunocompromised patients have been reported [27,28], as well as a case of pneumonia in which *Tritrichomonas* spp. was found in the bronchoalveolar fluid of a patient with AIDS [29]. In addition, squirrel monkeys and tree shrews are widely used as experimental models for human disease owing to their genetic background and physiological characteristics being so close to those of humans [30]. Recently, *T. batrachorum* was detected in feces samples of both NHPs and zookeepers, drawing attention to a possible zoonotic transmission [8]. Although there is no conclusive evidence of human infection with *T. mobilensis*, more attention needs to be paid to human clinical cases.

*Trichomonas* spp. infection is typically diagnosed by host specificity and parasitism site, as well as microscopic observation of the number of flagella [7]. However, the specific *Trichomonas* species cannot be identified by these methods; instead, molecular biology assays are used to differentiate among species. ITS1-5.8S-ITS2 gene sequence analysis is the most used method and relies on several known SNPs to distinguish specific species [31]. However, the present study on the presence of *T. mobilensis* in captive ring-tailed lemurs has several limitations. First, an objective assessment of environmental risks (shared water, mechanical vectors) is lacking. A second limitation is a lack of direct evidence of *T. mobilensis* transmission; infection experiments have not been conducted from squirrel monkeys to ring-tailed lemurs in the lab. Third, evidence of *T. mobilensis* PCR positivity in internal organs without histological lesions requires cautious interpretation. It is a clinical case; the samples in this study were not sampled precisely, the scissors and forceps were not replaced after sampling each organ, and the possibility that the internal organs have been contaminated by feces cannot be ruled out.

In conclusion, this is the first report of *T. mobilensis* isolation from ring-tailed lemurs, which expands the known host spectrum of the parasite beyond squirrel monkeys, tree shrews, and gibbons. Our findings suggest that environmental segregation and enhanced cleaning procedures should be implemented for squirrel monkeys raised in captivity to avoid feces-mediated *T. mobilensis* transmission, especially to ring-tailed lemurs.

## Data Availability

The original contributions presented in this study are included in the article. Further inquiries can be directed to the corresponding author.

## References

[1] Gerrick E.R., Howitt M.R. (2025). The lost kingdom: Commensal protists in the gut microbiota. Trends Microbiol..

[2] Li W.C., Ying M., Gong P.T., Li J.H., Yang J., Li H., Zhang X.C. (2016). *Pentatrichomonas hominis*: Prevalence and molecular characterization in humans, dogs, and monkeys in Northern China. Parasitol. Res..

[3] Zhang N., Zhang H., Yu Y., Gong P., Li J., Li Z., Li T., Cong Z., Tian C., Liu X. (2019). High prevalence of *Pentatrichomonas hominis* infection in gastrointestinal cancer patients. Parasit. Vectors.

[4] Chen D.Q., Wang Q.Y., Li Q.Q., Luo X.Y., Wu X.H., Wang J.P., Gao S.C., Liu X.C., Li W. (2024). The first report of *Tritrichomonas Foetus* and *Tetratrichomonas Buttreyi* in Raccoon Dogs (*Nyctereutes Procyonoides*) in China. Acta Parasitol..

[5] Ali S., Khetpal N., Khan M., Rasheed M., Asad-Ur-Rahman F., Echeverria-Beltran K. (2017). A Mexican honeymoon marred by gastrointestinal upset: A case of *Dientamoeba fragilis* causing post-infectious Irritable Bowel Syndrome. Cureus.

[6] Culberson D.E., Scimeca J.M., Gardner W.A., Abee C.R. (1988). Pathogenicity of *Tritrichomonas mobilensis*: Subcutaneous inoculation in mice. J. Parasitol..

[7] Bowman D.D. (2021). Georgis’ Parasitology for Veterinarians.

[8] Dib L.V., Barbosa A.D.S., Correa L.L., Torres B.D.S., Pissinatti A., Moreira S.B., Teixeira R.H.F., Costa A.L.M.D., Muniz J.A.P.C., Junglos A.M. (2024). Morphological and molecular characterization of parabasilids isolated from ex situ nonhuman primates and their keepers at different institutions in Brazil. Int. J. Parasitol. Parasites Wildl..

[9] Brack M., Kaup F.J., Fuchs E. (1995). Intestinal trichomoniasis due to *Tritrichomonas mobilensis* in tree shrews (*Tupaia belangeri*). Lab. Anim. Sci..

[10] Midlej V., Pereira-Neves A., Kist L.W., Bogo M.R., Benchimol M. (2011). Ultrastructural features of *Tritrichomonas mobilensis* and comparison with *Tritrichomonas foetus*. Vet. Parasitol..

[11] Culberson D.E., Pindak F.F., Gardner W.A., Honigberg B.M. (1986). *Tritrichomonas mobilensis* n. sp. (*Zoomastigophorea: Trichomonadida*) from the Bolivian squirrel monkey *Saimiri boliviensis boliviensis*. J. Protozool..

[12] Pindak F.F., Pindak M.M., Abee C.R., Gardner W.A. (1985). Detection and cultivation of intestinal trichomonads of squirrel monkeys (*Saimiri sciureus*). Am. J. Primatol..

[13] Scimeca J.M., Culberson D.E., Abee C.R., Gardner W.A. (1989). Intestinal trichomonads (*Tritrichomonas mobilensis*) in the natural host *Saimiri sciureus* and *Saimiri boliviensis*. Vet. Pathol..

[14] Zachary J.F. (2022). Pathologic Basis of Veterinary Disease.

[15] Felleisen R.S., Lambelet N., Bachmann P., Nicolet J., Müller N., Gottstein B. (1998). Detection of *Tritrichomonas foetus* by PCR and DNA enzyme immunoassay based on rRNA gene unit sequences. J. Clin. Microbiol..

[16] Kumar S., Stecher G., Tamura K. (2016). MEGA7: Molecular Evolutionary Genetics Analysis Version 7.0 for Bigger Datasets. Mol. Biol. Evol..

[17] Boggild A.K., Sundermann C.A., Estridge B.H., Lindsay D.S. (2002). Comparable growth of *Tritrichomonas mobilensis* in two commercially available culture media. J. Parasitol..

[18] Reinmann K., Müller N., Kuhnert P., Campero C.M., Leitsch D., Hess M., Henning K., Fort M., Müller J., Gottstein B. (2012). *Tritrichomonas foetus* isolates from cats and cattle show minor genetic differences in unrelated loci ITS-2 and EF-1α. Vet. Parasitol..

[19] Boncyk L.H., Brack M., Kalter S.S. (1972). Hemorrhagic-necrotic enteritis in a baboon (*Papio cynocephalus*) due to *Vibrio fetus*. Lab. Anim. Sci..

[20] Paixão T.A., Malta M.C., Soave S.A., Tinoco H.P., Costa M.E., Pessanha A.T., Silva R.O., Coura F.M., Costa L.F., Turchetti A.P. (2014). Hemorrhagic colitis associated with *Salmonella enterica* serotype Infantis infection in a captive western lowland gorilla (*Gorilla gorilla gorilla*) in Brazil. J. Med. Primatol..

[21] Uzal F.A., Navarro M.A., Li J., Freedman J.C., Shrestha A., McClane B.A. (2018). Comparative pathogenesis of enteric clostridial infections in humans and animals. Anaerobe.

[22] Pasternack M.S. (2002). Impact and management of *Campylobacter* in human medicine-US perspective. Int. J. Infect. Dis. Suppl..

[23] Burrough E.R. (2017). Swine Dysentery. Vet. Pathol..

[24] Brady A.G., Pindak F.F., Abee C.R., Gardner W.A. (1988). Enteric trichomonads of squirrel monkeys (*Saimiri* sp.): Natural infestation and treatment. Am. J. Primatol..

[25] Lymbery A.J., Morine M., Kanani H.G., Beatty S.J., Morgan D.L. (2014). Co-invaders: The effects of alien parasites on native hosts. Int. J. Parasitol. Parasites Wildl..

[26] Yao C. (2012). Opportunistic human infections caused by *Tritrichomonas* species: A mini-review. Clin. Microbiol. Newsl..

[27] Okamoto S., Wakui M., Kobayashi H., Sato N., Ishida A., Tanabe M., Takeuchi T., Fukushima S., Yamada T., Ikeda Y. (1998). *Trichomonas foetus* meningoencephalitis after allogeneic peripheral blood stem cell transplantation. Bone Marrow Transplant..

[28] Zalonis C.A., Pillay A., Secor W., Humburg B., Aber R. (2011). Rare case of trichomonal peritonitis. Emerg. Infect. Dis..

[29] Duboucher C., Caby S., Dufernez F., Chabé M., Gantois N., Delgado-Viscogliosi P., Billy C., Barré E., Torabi E., Capron M. (2006). Molecular identification of *Tritrichomonas foetus*-like organisms as coinfecting agents of human *Pneumocystis* pneumonia. J. Clin. Microbiol..

[30] Harding J.D. (2017). Nonhuman primates and translational research: Progress, opportunities, and challenges. ILAR J..

[31] Frey C.F., Müller N. (2012). *Tritrichomonas*–systematics of an enigmatic genus. Mol. Cell Probes.

